# Supplementary material for: Effects of research disturbance on nest survival in a mixed colony of waterbirds
Source: PeerJ. 2019 Oct 11;7:e7844. doi: 10.7717/peerj.7844 (PMC6791343; doi:10.7717/peerj.7844)
Supplement: Supplemental Information 1 [file peerj-07-7844-s001.pdf]

Supplementary material:

Model selection for differences among species in the two separate dataset (control vs. disturb zones)

We applied the following general model to the two separated dataset (disturbed zone and control zone)

$$log_e\left(\frac{s_{ijk}}{1-s_{ijk}}\right) = Day_{ijk} + Height_{ijk} + Species_{ijk} + Height_{ijk} \times Species_{ijk} + Year_j + Tree_{k(j)}$$

$$Year_j \sim N(0, \sigma_{Year}{}^2)$$

$$Tree_{k(j)} \sim N(0, \sigma_{Tree}{}^2)$$

where s_{ijk} is the daily survival rate of nest i during year j in tree k. Model selection is presented below for control zone (Table 1) and disturbed zone (Table 2).

(Intercept)	Day	Height	Species	Height x Species	K	Loge(L)	AICc	delta	weight
**4.4**	**-0.029**	**+**			**6**	**-228.5**	**469.1**	**0.00**	**0.41**
21.5	-0.031	+	+	+	12	-222.5	469.2	0.05	0.40
4.6	-0.031	+	+		8	-228.3	472.7	3.55	0.07
5.2	-0.024				4	-232.9	473.8	4.68	0.04
3.7		+			5	-232.2	474.4	5.26	0.03
20.7		+	+	+	11	-226.3	474.8	5.65	0.02
5.5	-0.027		+		6	-232.0	476.1	7.01	0.01
4.6					3	-235.5	476.9	7.79	0.01
3.8		+	+		7	-232.1	478.2	9.08	0.00
4.7			+		5	-235.1	480.1	11.00	0.00

Table 1. Model selection table to explain daily survival rate of Cattle Egrets, Little Egrets and Glossy Ibis nests in the non-disturbed zone. *K* is the number of parameters in the model, $Log_e(L)$ is the value of the maximized log-likelihood function, AIC_c is Akaike's Information Criterion adjusted for small samples, ΔAIC_c is the scaled value of AIC_c, and *weight* is the Akaike weight. Preferred model is presented in bold. Note that second model and sixth model are nearly unidentifiable.

(Intercept)	Day	Height	Species	Height:Species	df	logLik	AICc	delta	weight
**5.6**	**-0.04**	**+**	**+**		**8**	**-213.75**	**443.61**	**0**	**0.46**
6.3	-0.04		+		6	-215.89	443.85	0.24	0.41
20.1	-0.04	+	+	+	12	-211.50	447.24	3.63	0.08
4.8	-0.03	+			6	-219.43	450.93	7.32	0.01
5.5	-0.03				4	-221.47	450.97	7.36	0.01
5.0			+		5	-220.61	451.26	7.65	0.01
4.4		+	+		7	-219.04	452.16	8.55	0.01
4.7					3	-223.90	453.82	10.21	0.00
4.1		+			5	-222.30	454.65	11.03	0.00
19.2		+	+	+	11	-216.58	455.37	11.76	0.00

Table 2. Model selection table explaining daily survival rate of Cattle Egrets, Little Egrets and Glossy Ibis nests in the disturbed zone. *K* is the number of parameters in the model, $Log_e(L)$ is the value of the maximized log-likelihood function, AIC_c is Akaike's Information Criterion adjusted for small samples, ΔAIC_c is the scaled value of AIC_c, and *weight* is the Akaike weight. Preferred model is presented in bold. Note those third and last models are nearly unidentifiable.
